# Provider accountability as a driving force towards physician–hospital integration: a systematic review

**DOI:** 10.5334/ijic.1582

**Published:** 2015-03-31

**Authors:** Jeroen Trybou, Paul Gemmel, Lieven Annemans

**Affiliations:** Ghent University, Ghent, Belgium; Department of Management, Innovation & Entrepreneurship, Ghent University, Ghent, Belgium; Department of Public Health, Ghent University, Ghent, Belgium; Medicine and Health Sciences, Vrije Universiteit Brussel, Brussels, Belgium

**Keywords:** physician–hospital integration, provider financial risk bearing, methods, literature review

## Abstract

**Background:**

Hospitals and physicians lie at the heart of our health care delivery system. In general, physicians provide medical care and hospitals the resources to deliver health care. In the past two decades many countries have adopted reforms in which provider financial risk bearing is increased. By making providers financially accountable for the delivered care integrated care delivery is stimulated.

**Purpose:**

To assess the evidence base supporting the relationship between provider financial risk bearing and physician–hospital integration and to identify the different types of methods used to measure physician–hospital integration to evaluate the functional value of these integrative models.

**Results:**

Nine studies met the inclusion criteria. The evidence base is mixed and inconclusive. Our methodological analysis of previous research shows that previous studies have largely focused on the formal structures of physician–hospital arrangements as an indicator of physician–hospital integration.

**Conclusion:**

The link between provider financial risk bearing and physician–hospital integration can at this time be supported merely on the basis of theoretical insights of agency theory rather than empirical research. Physician–hospital integration measurement has concentrated on the prevalence of contracting vehicles that enables joint bargaining in a managed care environment but without realizing integration and cooperation between hospital and physicians. Therefore, we argue that these studies fail to shed light on the impact of risk shifting on the hospital–physician relationship accurately.

## Introduction

In many countries, integrated health care delivery plays an increasingly important role in current health care reform [1]. Hospitals and physicians lie at the heart of our health care delivery system. Both have been working together for years in providing secondary health services to the community. While physicians provide and coordinate the care, the hospital provides the resources in which that care can be managed and delivered [2]. Consequently, it has been argued that the relationship between the medical specialist and the hospital has an influence on the quality of provided care and cost-effectiveness of health care delivery [3]. Currently, many western countries are seeking ways to increase provider accountability by installing provider financial risk bearing for the delivered care [4]. These efforts reflect stakeholders’ expectations of improving performance in response to two important evolutions. On the one hand, there is recognition that health care systems are fragmented and suffer from unexplained variability and gaps in quality of care [5]. On the other hand, rising health care expenditures are a global phenomenon. This trend is likely to increase further, following the recession that became widespread since 2009 [6]. The confluence of these forces makes it unlikely that hospitals or physicians will be able to meet these challenges without increased collaboration and closer integration [7]. It is important that in this view, physician–hospital integration is not seen as an end in itself but rather as a means for improving cost-effective performance of secondary care and as a precondition for the creation of added value for the patient and society. The aim of this paper is to provide insight into the relationship between provider accountability and physician–hospital integration and assess the evidence base. We applied the principles of agency theory and developed a conceptual framework to increase our understanding of this relationship. We continue with a systematic review of the literature and a discussion of the methods used to measure the concept of physician–hospital integration.

## Theory and methods

### Theoretical framework

To increase our understanding of the hospital–physician relationship we build on agency theory. The concepts of agency theory have been found highly applicable in discussing health care [8,9]. The agency dilemma is present when one party delegates work to another, who performs the work. The ‘principal’ invokes an ‘agent’ with specialized skills or knowledge to perform the task in question. An agency problem occurs when the agent does not have exactly the same objectives or motivations as the principal and does not necessarily act in the best interest of the principal [10].

**Figure 1.**
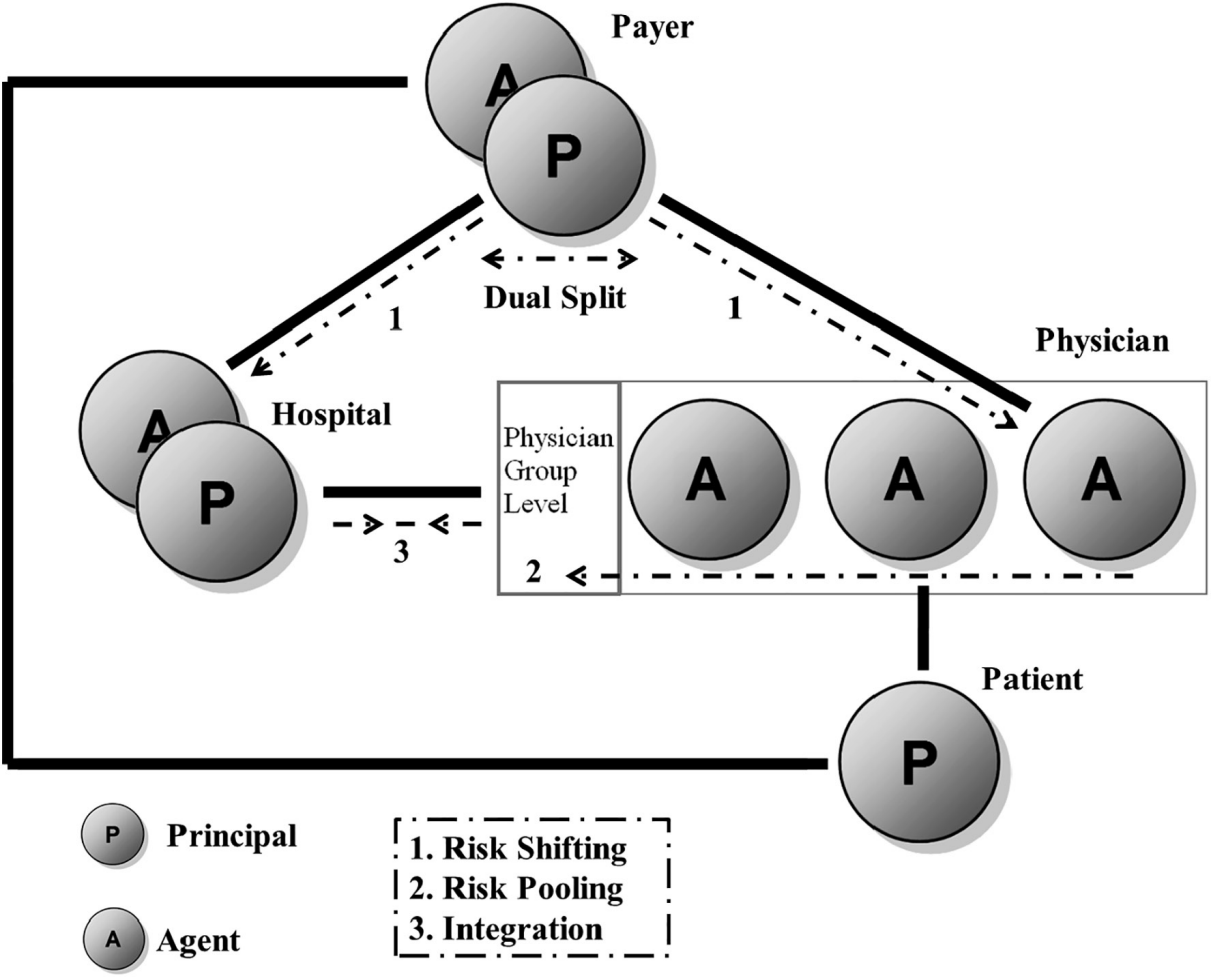
An agency perspective of the hospital–physician relationship.

As depicted in Figure 1, secondary care is characterized by several interdependent agency relationships. First, the fiduciary relationship between the medical doctor and the patient lies at the heart of secondary care. In this relationship physicians use their competence in the individual patient's best interest [11]. Second, health insurers act as agent for the patients or population as a prudent buyer of care on behalf of the consumers. Following Boadway and colleagues [12], we make abstraction from these two relationships (patient–physician and patient–payer) by assuming that they are passive to the risk distribution problem occurring in the hospital–physician relationship. Finally, a two-tier hierarchy of principal-agent interactions in hospital care delivery can be identified [8]. The top one involves the payer as principal to the hospital and physician; the second one involves the hospital as principal to the medical staff. A dual split in payment is made in which physicians and hospital have their own distinct compensation scheme. In this setting, physicians act as independent care givers generating medical fees and other operating expenses are covered by a hospital budget.

In this study, we concentrate on these three important relationships. More precisely, the payment framework with the associated financial incentives is used as an instrument to attain the goal of physician–hospital integration. Specifically, we argue that within the agency framework three important processes can be identified: (a) risk shifting from payer to providers, (b) risk pooling within physician groups and (c) integration between the hospital and the medical staff.

### Risk shifting towards providers

Integrated health care delivery plays an increasingly important role in current health care reform efforts [13]. Moreover, many countries have adopted reforms in which providers are made financially accountable for the delivered care. This process has been referred to as ‘risk shifting’ towards providers [2]. As new payment methods have emerged, the nature of the underlying parameters changed, resulting in a varying financial risk allocation between payer and providers. Traditionally, physicians were paid on a fee-for-service basis and hospitals were reimbursed for the costs incurred [14]. As such, the financial risk associated with secondary care delivery was retained at the payer level. However, this situation has changed in recent decades. More specifically, payment systems have evolved from a retrospective, cost-reimbursement to prospective financing systems, making providers partly accountable for their expenditures. In these prospective payment systems, the provider's payment rates or budgets are no longer directly linked with the individual costs or efforts of the provider, introducing a certain financial risk at the provider level [15]. In addition, recognition that the health care system suffers from gaps in quality and safety has stimulated a broad array of initiatives to improve performance by fostering greater accountability from the part of providers and the development of value-based purchasing [16].

#### Risk pooling in physician groups

Physicians usually operate as quasi-independent professional agents in a physician group setting. These structures that foster shoulder-to-shoulder practice function as financial intermediaries between the payer and the individual physician [17]. In this setting, two tiers of financial incentives bear on physician behaviour: the method of payment by the payer and the method used by the medical group to compensate individual physicians. As the individual physicians are sometimes paid on a different basis than the group, a risk adjustment can be made at the individual practitioner level. For instance, while the reimbursement system of physicians could rely on capitation (with a fixed fee per capita), these financial means can be pooled at the physician group level (pooling the fees of all the physicians belonging to that physician group) and an alternative remuneration system for the individual physicians can be applied (e.g. a fee per patient visit instead of a fee per patient). While in this exemplary case the physician group is reimbursed by capitation, the individual physicians are compensated (and incented) by an alternative compensation system. This intermediate structure thereby changes the financial risk bearing of the individual physicians. Thus, risk assumption may operate at different levels in organizational settings, the first via a group effect and the latter at the individual physician level.

### Physician–hospital integration

Since the initiation of prospective payment, hospitals have been struggling to develop strategies that improve their prospects for long-run financial viability. Given physician autonomy, the most important one has been the effort to build effective hospital–physician relations [18]. This effort has been described as physician–hospital integration. Previous research has identified three types of integrative actions [19]. Although these types were initially conceptualized in the context of physician linkages to health plans, it has been demonstrated empirically that the categories of integrative actions apply also to the hospital setting [20,21]. First, physician–system integration is the extent to which physicians are economically linked to a system, use its facilities and services and participate actively in its planning, management and governance. Second, functional integration is defined as the extent to which key support functions and activities are coordinated across operating units to add the greatest overall value to the system. Third, clinical integration encompasses hospitals’ structures and systems to coordinate patient services across people, functions, activities and sites over time [22]. Common examples of clinical integration are clinical pathways or interdisciplinary electronic patient records. Based on the findings of these researchers and drawing on academic and consulting literature, Burns and Muller [20] proposed an alternative, improved classification of hospitals’ efforts to align their medical staff. Besides clinical integration, these researchers make a distinction between economic integration, referring to the contractual, monetary relationship between both and noneconomic integration, emphasizing the cooperative nature needed in their day-to-day relationship.

### Search strategy

**Figure 2.**
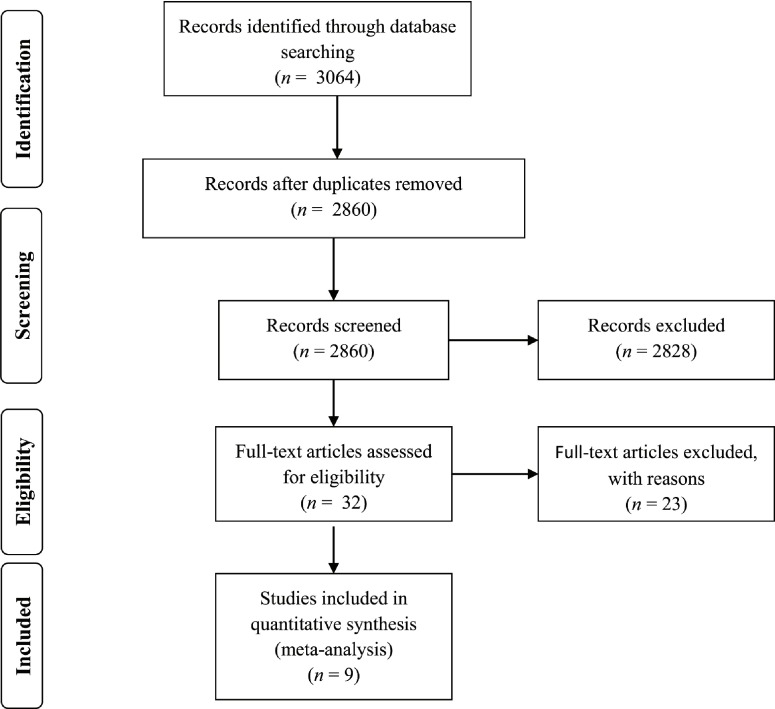
Flow diagram.

Electronic databases (Medline, CINAHL, Web of Science, EconLit and EBSCO) were searched in June 2013 for studies focusing on the relationship between provider financial risk bearing and physician–hospital integration by the development of key search terms. The final search pattern was: [(Salaries and Fringe Benefits OR Reimbursement OR Incentive OR Fees and Charges OR pay* OR incentive* OR compensation* OR reimbursement* OR financ* OR bonus* OR remunerat*) AND (hospital AND physician) AND (integration OR relation* OR alignment)]. In addition, reference lists of all included papers were further examined and additional articles were retrieved. We restricted the studies eligible for inclusion to those published in peer-reviewed journals in English between January 1989 and June 2013. This time frame was selected because in this period new organizational arrangements with tighter affiliation between physicians and hospitals were initiated in the USA [23]. In the same period health care policy debate in European countries also concentrated on the pros and cons of introducing some form of ‘managed competition’ or ‘internal markets’ to enhance efficiency of health care delivery and to contain costs [24]. A first selection was made on title and abstract. All key articles that were potentially useful to this review were identified. Afterwards, each article was fully read and judged on relevance. Finally the articles were narrowed down according to the inclusion and exclusion criteria. The inclusion criteria stipulated that citations should be: a peer-reviewed English journal, across USA or Europe and be conceptual, quantitative or qualitative. The exclusion criteria stipulated that citations cannot be: industry extracts or scholarly publications focusing the relation between hospitals and primary care physicians. Abstracts of relevant citations were read and classified in two categories (directly relevant and not relevant). Only the relevant citations explicitly focusing on the link between provider financial risk bearing and physician–hospital integration were included for the review. In total, 3064 studies were identified (204 duplicates) and ultimately 9 studies which explicitly focused on the relationship between provider accountability and physician–hospital integration were included in this review. Figure 2 provides a schematic overview.

## Results

We identified nine scientific papers that fulfilled the selection criteria. They are presented in Table 1. Our systematic review indicates that previous research has largely focused on describing the formal structures of different physician–hospital organizational arrangements and assessing their prevalence in the advent of managed care. Although it has been demonstrated that the most these organizations occurred in markets where managed care grew rapidly [24], only one empirical study was able to demonstrate that managed care is a driving force towards integration [25]. The other studies conclude that the explanatory power is weak [26].

Our review shows that a methodological critique of the literature is highly needed. Moreover, previous studies have used different and inadequate measurements of both physician–hospital integration as well as provider financial risk bearing. This stresses the need of a methodological critique which remained absent in the literature.

## Discussion

### Physician–hospital integration

Previous studies have focused predominantly on the intermediary organizational models between physicians and hospitals. While these structures could be a step to increased collaboration, it can be argued that simply creating structures does not guarantee achievement of true integration. More precisely, the literature has largely focused on describing the formal structures of different physician–hospital organizational arrangements as an indicator of physician–hospital integration [22–24]. Table 2 provides an overview of these formal arrangements.

Although data on the contractual relations between physicians and hospitals are readily available and therefore relatively easy to capture, we have some concern regarding the true measurement of integration. While the intermediary organizational models are a step to increased collaboration, it can be argued that simply creating structures does not guarantee achievement of true integration. More specifically, the danger exists of creating a contracting vehicle with the sole purpose of joint bargaining in a managed care environment without realizing true cooperation and integration [22]. Physician–hospital integration is clearly more than just strengthening the economic ties between both. Instead, from a policy perspective, added value is realized by increasing the underlying day-to-day cooperation in order to improve efficient care delivery and to improve the quality of the delivered care. This is congruent with the fact that only limited differences in the degree of underlying integrative processes across the different organizational models can be identified [25]. Given these difficulties concerning the measurement of true integration by means of contractual arrangements between physicians and hospitals, the alternative approach of concentrating on the underlying processes of integration (the increased cooperation that leads to added value) is a promising line of research. In addition, since health policy reform focuses on gradually introducing additional incentives tied to outcomes of care (i.e. pay for performance), it would be valuable to study the impact on the clinical dimension of physician–hospital integration.

### Provider financial risk bearing

Our theoretical framework has shown that the economic relationships between the payer, hospital and physician(s) are highly complex. However, most studies have measured provider financial risk bearing solely by the advent of managed care [22,26]. In this umbrella concept a variety of payment arrangements are used including not only capitation but also discounted fee-for-service or case-based payments. Future research should therefore focus on the financial risk installed at the provider level by the underlying payment mechanisms. In addition, our results show that previous studies have measured provider risk bearing in a fragmented way. Consequently, these studies fail to shed light on the impact of risk shifting on the hospital–physician relationship accurately. First, because of the dual split in payment and physician autonomy in medical decision-making, the degree of risk assumed by the hospital also depends on the alignment of incentives with the medical staff [21]. More specifically in the situations in which the hospital bears a certain degree of financial risk (e.g. per case payment) and the medical staff's financial responsibility for their actions remains obsolete or limited (e.g. fee-for-service) the hospital's risk can be considerably increased. Therefore, besides the financial risk induced by the payment scheme, the degree of alignment between the separate revenue streams of the hospital and the medical staff should be included. Second, the financial relationships between physicians (risk pooling) and physicians with their hospital (risk sharing) should be considered. More precisely this instals the possibility to share the risk induced by the payment framework between providers. The economic relationships between providers can be used as an instrument to realize this risk sharing. Two possibilities exist. On the one hand the contractual relationship between physicians and the hospital they practice at can be used as an instrument to align incentives and share risk between medical staff and hospital [20]. On the other hand, the physician group level can function as the financial intermediary between the payer and the individual physician resulting in risk pooling between physicians [33].

Finally, current health policy reform concentrates on the introduction of incentives tied to quality of the delivered care. It is therefore surprising that up to now no empirical research studies have studied the impact on physician–hospital integration. Moreover, since most countries have introduced this payment mechanism in a progressive way (by gradually increasing the scope of the programme and the size of payments) this encompasses a promising opportunity to study this in a longitudinal way.

### Limitations

Although payment reform has been identified as one of the main driver to new hospital–physician relationships, we note that besides provider financial risk bearing also other market forces which were not included in our study could be potentially important to physician–hospital integration.

First, the recognition that the health care system suffers from serious gaps in quality (e.g. medical errors, unnecessary differences in practice patterns and unintended variation in outcomes) has stimulated a broad array of public- and private-sector initiatives to improve performance [34]. More precisely, accreditation and public reporting of hospital quality have become the locus of have emerged as advocated strategies [35] and as a result, physician–hospital integration is stimulated.

Second, the level of competition has been identified as one of the other main forces that impact hospitals [36]. Moreover care that has historically been delivered in a hospital inpatient setting can increasingly be performed in a short-stay or even ambulatory setting. Consequently, beside the traditional full-service general hospital, specialized facilities owned by physicians have emerged as alternative settings of care delivery [37]. This could have a negative impact on physician–hospital integration.

## Conclusion

This paper addressed the study of provider financial risk bearing as a driving force towards physician–hospital integration. The previous sections have shown that increasing the accountability for the provided care theoretically enhances physician–hospital integration. Ultimately, this integration is considered to be a precondition for the creation of added value for the patient and cost-effective care delivery. Our findings, however, can at this time be supported merely on the basis of the theoretical insights of agency theory rather than empirical research. More precisely agency theory proposes that a greater degree of financial risk bearing instal a greater degree of interdependency and thereby induces a greater need for physician–hospital integration. However, this theoretical insight is not adequately supported by empirical evidence. Two methodological issues contribute to this result of our systematic review. First, previous research has measured provider risk bearing in a fragmented way. Second, physician–hospital integration measurement has concentrated on the prevalence of contracting vehicles that enables joint bargaining in a managed care environment but without realizing integration and cooperation between hospital and physicians. Therefore, we argue that these studies fail to shed light on the impact of risk shifting on the hospital–physician relationship accurately. However, we opened up a point of departure for studying the role of provider financial risk bearing in physician–hospital integration in greater depth. From this starting point, additional research needs to be executed.

## Figures and Tables

**Table 1.**
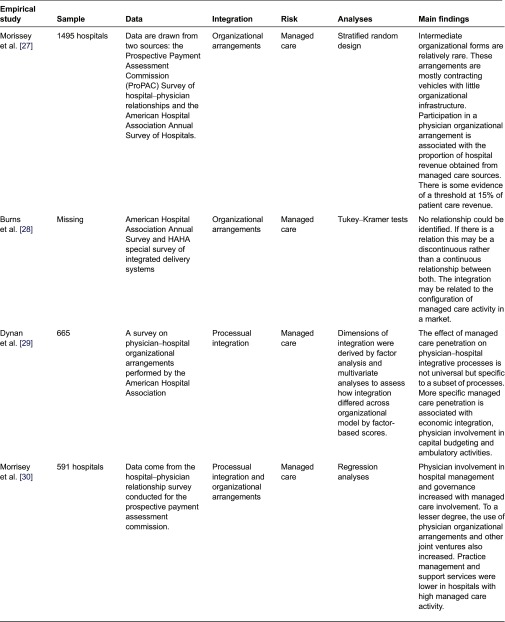

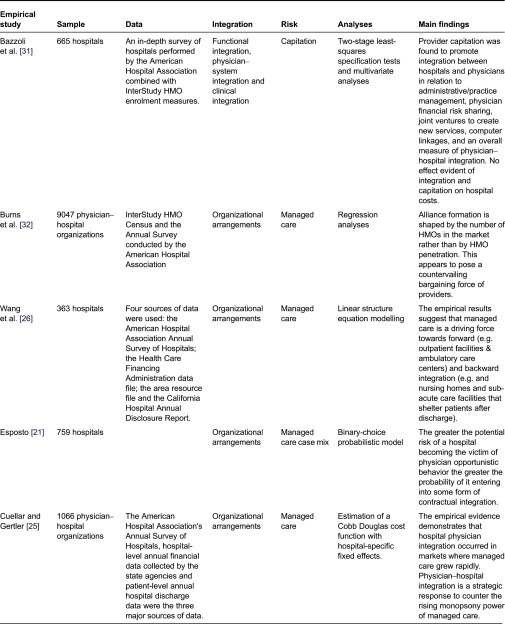
Studies included in the systematic review

**Table 2.**
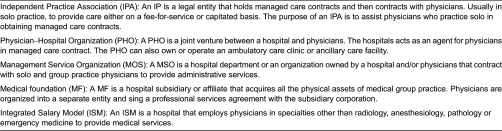
Definitions of physician–organizations arrangements

Dynan et al. (1998).
